# A Qualitative Process Evaluation of Classroom-Based Cognitive Behaviour Therapy to Reduce Adolescent Depression

**DOI:** 10.3390/ijerph110605951

**Published:** 2014-06-05

**Authors:** John A. Taylor, Rhiannon Phillips, Ellen Cook, Lucy Georgiou, Paul Stallard, Kapil Sayal

**Affiliations:** 1 Division of Psychiatry and Applied Psychology, Institute of Mental Health, University of Nottingham, Triumph Road, Nottingham NG7 2TU, UK; E-Mail: kapil.sayal@nottingham.ac.uk; 2 Wales School for Primary Care Research, Institute of Primary Care and Public Health, School of Medicine, Cardiff University, 5th Floor, Neuadd Meirionnydd, Heath Park, Cardiff CF14 4YS, UK; E-Mail: PhillipsR19@cardiff.ac.uk; 3 Department for Health, University of Bath, 22–23 Eastwood, Bath BA2 7AY, UK; E-Mails: ellencook62@hotmail.com (E.C.); lucygeorgiou@hotmail.com (L.G.); p.stallard@bath.ac.uk (P.S.)

**Keywords:** depression, adolescents, prevention, school, programme, evaluation, qualitative

## Abstract

Small scale trials indicate that classroom-based Cognitive Behaviour Therapy (CBT) for adolescents has good reach and can help prevent depression. However, under more diverse everyday conditions, such programmes tend not to show such positive effects. This study examined the process of implementing a classroom-based CBT depression prevention programme as part of a large (n = 5,030) randomised controlled trial across eight UK secondary schools which was not found to be effective (PROMISE, ISRCTN19083628). The views of young people (n = 42), teachers (n = 12) and facilitators (n = 16) involved in the Resourceful Adolescent Programme (RAP) were obtained via focus groups and interviews which were thematically analysed. The programme was considered to be well structured and contain useful content, particularly for younger pupils. However, challenges associated with implementation were its age appropriateness for all year groups, its perceived lack of flexibility, the consistency of quality of delivery, the competing demands for teacher time and a culture where academic targets were prioritised over personal, social and health education. Whilst schools are convenient locations for introducing such programmes and allow good reach, the culture around improving well-being of young people in schools, increasing engagement with teachers and young people and sustaining such programmes are issues that need addressing.

## 1. Introduction

The incidence of depression rises sharply in teenage years [1] and it is estimated that up to 10% of young people will have experienced a clinically significant depressive disorder by age 16 [2]. Depression in adolescence is associated with impaired academic performance, social difficulties, substance misuse and self-harm, yet often goes unrecognised and untreated [3,4]. Therefore, depression in adolescence is a major public health issue and preventative interventions need to be explored.

Schools are viewed as important settings for implementing depression prevention programmes as they enable large numbers of young people to be reached at a time when they are vulnerable to the onset of depression [5,6,7]. Compared to targeted interventions, universally delivered depression prevention programmes have good reach, reduce possible negative effects of stigma and result in lower rates of dropout [8,9,10,11]. Whilst universal approaches typically have a more limited effect at an individual level than targeted approaches, they have the potential to prevent more disorders at the population level [12].

A recent systematic review of school-based prevention and early intervention programmes for depression [5] has provided some support for both targeted and universal interventions. Half of the trials in this review reported significantly reduced symptoms of depression following the intervention, ranging from small (0.21) to large (1.40) effect sizes. These findings are consistent with evaluations that have been carried out of targeted and/or universal classroom-based mental health-related prevention programmes using Cognitive Behavioural Therapy (CBT) principles, including; the Penn Resiliency Programme [13,14], Coping with Stress [8], Problem Solving for Life [11], FRIENDS [15], Resourceful Adolescent Programme [10] and other CBT programmes [16]. The Resourceful Adolescent Programme (RAP) has been relatively well evaluated and there is evidence for its efficacy in the short to medium term when delivered in school settings by outside interventionists or existing teachers [17,18]. For RAP, greater reduction in symptoms of depression relative to non-intervention control groups have been reported, and good reach (>70% of the eligible population) and low attrition (<10%) can be achieved [9,10,17]. 

Despite being a promising approach, some larger scale studies have recently failed to find an effect of universally delivered depression prevention programmes in schools. For instance, a large scale trial (n = 5,633) of Beyondblue, an intervention delivered by trained and supported teachers, was carried out in Australia and failed to show effectiveness in preventing depression [19,20]. Further, in a recent evaluation of the UK Resilience Programme with Year 7 students across 22 schools under everyday conditions, no sustained effect of the programme was found at one year [21]. In an effectiveness study in England, the Promoting Mental Health in Schools through Education (PROMISE) Project [22] health-led classroom-based CBT, in the form of RAP, was rolled out across several schools and age groups as part of a large scale randomised controlled trial (n = 5,030). Due to the universal nature of the delivery RAP had good reach and low attrition, and high treatment fidelity was demonstrated [23]. Nonetheless, no effect of classroom-based CBT was found on symptoms of depression relative to usual school provision or attention control conditions [23]. 

One possible explanation for such findings is that programme effects are diluted when rolled out under more diverse everyday conditions. However, the reasons why this might occur when using a manualised programme with good reach and treatment fidelity are unclear. Other reasons could be due to a lack of specific training, knowledge and experience in delivering the programme, or group leaders’ social competence, motivation towards the programme and the quality of supervision [24,25]. Qualitative process evaluation can be useful in understanding outcome results in trials of complex interventions [26].

Previous research has shown a number of challenges associated with implementing mental health interventions in schools, such as limited time being made available for emotional/mental health education, lack of training, insufficient time to implement and plan programmes, and difficulties in engaging with recipients and agreeing expectations [19,27,28,29,30]. More specifically, mental health promotion in schools continues to be a secondary priority over academic attainment [31], and teachers do not receive sufficient training in the delivery of Personal, Social and Health Education (PSHE) [32]. Engagement in such programmes is likely to be strongly influenced by levels of teacher and pupil satisfaction [33]. A number of studies have obtained feedback following relevant initiatives. For example, teachers who delivered the Aussie Optimism Programme to 11-13 year olds found the content appropriate and felt confident to deliver it [34]. They reported increased confidence in pupils following participation and a greater ability to manage interpersonal conflict. Similarly, after completing the Problem Solving for Life Programme with 12–14 year olds, a high percentage of the teachers felt the course had been effective and stated that they would teach the course again in the future [11]. However, half of the teachers had insufficient time to complete all the tasks. Teachers delivering RAP-Kiwi to 13–15 year olds felt that they could have done so more effectively if they had been able to deviate from the manual to better suit individual classes and adapt the way some of the concepts were taught [9]. Pupils aged 12–14 who participated in the Problem Solving for Life Programme reported improved perceptions in their ability to solve problems after the intervention [11], but only 34% felt they would utilise the skills learnt in the future. More than half of the pupils who provided evaluation data about their participation in RAP in Canada [18] stated that it had helped them to manage conflicts, deal with stress and be more positive and self-confident. Despite such findings, in-depth qualitative process evaluations of complex depression prevention programmes in schools are scarce [35]. Thus, insights about the perceptions of providers and recipients and how these might influence successful implementation are limited.

The current study is a qualitative evaluation that was carried out as part of the PROMISE Project. The aims were to investigate the perceptions of facilitators, teachers, and young people of the process of a classroom-based CBT depression prevention programme in schools. Such an approach is important for exploring how an intervention is implemented and received in its setting and assists in interpreting its outcomes [26].

## 2. Method

### 2.1. Research Design

The qualitative evaluation of the PROMISE Project involved semi-structured interviews or focus groups with intervention facilitators, teachers and young people post-intervention. The majority participated in focus groups to enable discussion and exchange of views within the groups. One-to-one interviews were carried out with teachers when only one representative was available to provide qualitative feedback from a school. The advantages of using focus groups and interviews are that they allow participants to respond in their own words and for researchers to ask questions to uncover reasons for responses which might not be revealed using survey methods.

### 2.2. Context

The PROMISE Project was a large scale randomised controlled trial (n = 5,030) carried out in eight schools in the South West and East Midlands regions of England. The programme was first piloted in one different school [36]. The study investigated the effectiveness and cost-effectiveness of a universally delivered school based programme in preventing depression in high risk adolescents aged 12–16 years. High-risk status was determined by scores on both the screening and baseline short-form Mood & Feelings Questionnaire (SMFQ) [37] and data about high-risk adolescents were analysed separately. The trial had three arms; RAP delivered by two facilitators external to the school with the teacher present to assist with classroom management (classroom-based CBT), the standard school PSHE curriculum delivered by teachers (usual school provision), and usual PSHE delivered by the teacher in conjunction with additional support from two facilitators external to the school (attention control). All facilitators had a minimum of an undergraduate university degree in a relevant discipline and an appropriate professional background or experience of working with children or young people. All were provided with initial training in the delivery of RAP and received on-going supervision.

The classroom-based CBT programme, RAP [17] is underpinned by CBT and interpersonal therapy principles, and comprised nine 40–50 min sessions delivered flexibly to fit in with existing school structures. Sessions covered stress management, self-esteem, affect regulation, cognitive restructuring, problem solving, conflict resolution, interpersonal skills and social networks. These areas were explored through multiple teaching methods such as psycho-education, skills building exercises, and role-plays [18].

### 2.3. Participant Recruitment

Ten year groups were randomised to classroom-based CBT across the eight schools of varied socio-economic status across different geographical regions. This comprised 79 classes where 73 class teachers were involved in the study and 40 facilitators delivered the classroom-based CBT. A purposive approach to sampling was adopted to obtain a cross-section of those involved in the classroom-based CBT by school/geographical region, role and responsibilities (teacher), and gender and age (young people). Young people were invited to take part in the focus groups via their teachers. The school link person (PSHE coordinator or member of the senior management team), PSHE class teachers, and intervention facilitators were invited to take part by the research team. The overall sample size was dictated by practical considerations, such as participant availability and time constraints. Participants were therefore largely self-selecting. Demographic characteristics across the 12 focus groups and 5 interviews are provided in Table 1.

**Table 1:** Demographic characteristics and group composition.

Focus Group/ Interview Type	Gender	School Code	Initials of Member of Team Who Coded Transcript
Young people (Year 8)	6 male	103	JT/EC/LG
Young people (Year 8)	6 female	103	JT
Young people (Year 8)	6 male/5 female	106	EC
Young people (Year 9)	1 male/4 female	102	LG
Young people (Year 9)	4 male/2 female	105	JT
Young people (Year 10)	2 male/4 female	105	JT
Young people (Year 11)	2 female	102	LG
Teacher	1 male	101	JT
Teacher	1 female	102	JT/EC/LG
Teacher	2 female	103	JT
Teacher	1 male/4 female	105	JT
Teacher	1 female	106	LG
Teacher	1 male	107	LG
Teacher	1 male	108	EC
Facilitator	1 male/7 female	South West	EC
Facilitator	3 female	South West	JT/EC/LG
Facilitator	5 female	East Midlands	JT

Notes: Age range: Year 8—12–13 years; Year 9—13–14 years; Year 10—14–15 years; Year 11—15–16 years.

### 2.4. Flexible Topic Guides

Separate semi-structured interview guides for pupil, teacher and facilitator groups were devised to capture in-depth information about key topics of interest and used flexibly to allow participants to discuss issues that were salient to them. The pupil guide assessed their overall impressions of RAP and their use of the skills learnt. The teacher guide sought their overall impressions of RAP, their views about the individual sessions and delivery of RAP and issues around maintenance and costs of RAP. This guide also assessed their attitudes towards PSHE more generally. The facilitator guide included overall impressions of the project content, views about delivery, pupils’ perceptions of the experience, training and supervision and long-term delivery of the programme.

### 2.5. Procedure

Interviews or focus groups lasted approximately one hour and took place between March and July 2010 after delivery of the programmes had been completed in each school and before findings of the trial were known. Each was conducted by the Trial Manager or post-doctoral Research Officers at each site, with a note taker present in the focus groups to aid subsequent transcription. 

### 2.6. Ethical Considerations

Ethical approval for the PROMISE Project was granted by the University of Bath School for Health Research Ethics Panel. Participants provided written informed consent. Confidentiality was assured and audio data were anonymised during transcription. Participants were asked to keep discussions within the group confidential and were reminded of their right to withdraw at any time.

### 2.7. Analysis

Interviews and focus groups were digitally audio-recorded and transcribed verbatim. NVIVO 9 software was used to aid analysis. Transcripts were thematically analysed by three members of the research team (JT, EC & LG) broadly following the guidelines of Braun and Clarke [38]. This comprised: (1) becoming familiar with the data; (2) generating initial codes; (3) searching for themes; (4) reviewing themes, and; (5) defining and naming themes. Based on the study’s main lines of enquiry and the issues arising from the transcripts, the research team reached consensus on a broad and descriptive coding framework to enable the generation of initial codes. Each coding unit was coded exclusively into just one category to provide clearly defined coding categories [39]. Coding was conducted at both a semantic level, *i.e.* from explicit or surface meanings of the data [38] and a latent level, *i.e.*, from underlying, implicit references to semantic content [39]. 

Validity and reliability are pertinent in qualitative inquiry [40]. Our verification strategy involved the three researchers firstly all coding three randomly selected transcripts. Inter-rater agreement ranged from 78%–100%, indicating satisfactory consistency. Any coding inconsistencies were resolved by discussion and consensus. Researchers coded the remaining transcripts individually using the same framework criteria, but with flexibility within this to fit with their data (e.g., creating sub categories or collapsing and renaming categories). Data were then reviewed by the researchers together and themes redefined as considered appropriate. The emergent inductively coded themes were examined to establish key facilitators and barriers to implementation of the classroom-based CBT. 

## 3. Results and Discussion

Final salient sub-themes emerging from the data were: types of teaching, useful aspects of the programme, number of sessions, quality of delivery, classroom management, rapport with young people, age, involvement of teachers, resources, future use of the programme, value of PSHE and busy school environment (see Table 2). To assist in organising the data, these sub-themes have been reported as appropriate under the following broader theme headings: the structure and content of the classroom-based CBT, the way in which it was delivered, whether it was sufficiently flexible and differentiated, whether it would be sustainable, and the challenges of implementing it in the school context.

**Table 2:** Summary of main themes.

Main Theme	Sub-themes	Key Points
Structure and content of the classroom-based CBT	Types of teaching	Hands-on activities preferred.
	Useful aspects of the programme	Useful aspects of the programme were highlighted, e.g., sessions on resolving conflict, recognising body signals, and problem solving.
	Number of sessions	Shorter faster-paced programme would have been preferred.
Delivery	Quality of delivery	Variable. Experience, confidence, reliance on scripts, and teacher engagement were important.
	Classroom management	Teachers and facilitators found this challenging and were unsure of their roles.
	Rapport with young people	Success in achieving this was variable, particularly where classes did not have the same facilitators throughout the programme.
Flexibility and differentiation	Age	The classroom-based CBT used (RAP) seemed to be more appropriate for Year 8 than older year groups.
	Involvement of teachers	Teachers wanted more flexibility and involvement in development of the classroom-based CBT.
Sustainability	Resources	Cost and time involved meant classroom-based CBT would not be sustainable in current form
	Future use of the programme	Teachers generally felt they could deliver the programme alone with the right training, although they would be most likely to adapt it and select some parts only.
Implementation in the school context	Value of PSHE	PSHE was perceived to be undervalued and under-resourced.
	Busy school environment	Insufficient lead in time, communication within schools, lack of time for contact between teachers and facilitators, were problematic.

### 3.1. Structure and Content

The classroom-based CBT programme (RAP) includes a number of different educational methods, with the aim of making the programme interesting and engaging for adolescents. This includes lecture-style delivery, small group discussions, use of multi-media presentations (e.g., video clips and images), and hands-on interactive activities such as role play and group tasks. 

Many facilitators reported that students typically related well to discussion topics, such as arguments with parents. However, they felt some concepts could have been better absorbed with greater use of interactive approaches, and in general, young people preferred the hands-on activities to discussion:

*I preferred doing the hands-on stuff... the discussions, sometimes I think they kind of just went off a bit because some people lost concentration.* (Year 11 female)

Some teachers also noted that the role plays and faster-paced interactive sessions were the most memorable:

*Being more interactive and getting the children involved a lot more.* (Teacher)

Some young people expressed a desire for more drama-related sessions and use of other electronic resources, such as interactive white boards and laptops. 

Several positive aspects of specific sessions were highlighted by young people and teachers, for example the sessions on seeing other people’s points of view using visual illusions and recognising physical signs of depression and anxiety:

*You’ve got a really good little connect activity actually here, this ‘two sides’ of things... and they love that.* (Teacher)

*I thought the body signals one was handy... I think that’s something that works across the age range.* (Teacher)

A Year 8 teacher was heartened by the students’ realisation that they had some control over their ways of thinking:

*You could see the light going on for so many of them… it was almost like it was news to them… that we don’t have to think like this. I saw it happening with a lot of them that they embraced it… you could see it working.* (Teacher)

The classroom based CBT consisted of 9 sessions as it has been suggested that programmes comprising 8 to 12 sessions are most likely to be effective perhaps because they offer sufficient time for individuals to assimilate the programme materials without becoming overloaded [5]. However, some facilitators and teachers felt that there was not enough content in particular sessions to fill the time available and that a shorter programme might have been more engaging:

*For the amount of work that was got through, I think it could’ve been condensed to four or five weeks.* (Teacher)

Certain teachers believed it would be feasible to combine some of the sessions to produce a more concise and tightly packed programme. Some pupils also commented that there was too much repetition in some of the sessions:

*Many of the lessons, we’d go in, learn about one thing, come out and the next lesson we’d go back in and we’d learn about exactly the same thing for about half an hour, and then we’d move on to a different thing*. (Year 9 male)

One pupil questioned whether it would be more effective to intersperse specific classroom-based CBT sessions with other activities across the yearly PSHE curriculum.

### 3.2. Delivery of Programme

A number of teachers reported that the quality of delivery of RAP was variable:

*Some of the people who delivered were quite comfortable and I felt quite successful in what they did. Whereas I got the impression from some of my colleagues that some of the people who came to teach it struggled really, and found it hard to deliver the materials in the way they needed to be delivered.* (Teacher)

Some teachers and pupils felt that some facilitators were over-reliant on their notes, which disrupted flow and had a negative influence on pupils. Facilitators generally reported finding it easier to deliver classroom-based CBT after a period of familiarisation:

*It definitely got easier the more you did the sessions because you just obviously knew them more by heart and didn’t have to rely on the script so much and you can just take the lead.* (Facilitator)

Most facilitators felt that active teacher engagement and support was imperative for a successful session:

*That made such a crucial difference with the teacher’s attitude, just… make or break… whether it went… how the class reacted to it.* (Facilitator)

A few facilitators commented on the negative attitudes of some teachers towards classroom-based CBT and the staff delivering the programme, which undermined their confidence to deliver sessions.

The relationship between teachers and facilitators, particularly around classroom management, was a frequently reported issue. Some teachers were mindful not to undermine the facilitators delivering classroom-based CBT and found it difficult to know when it was appropriate to intervene to improve classroom behaviour:

*I sometimes felt it was hard to know whether to, when to intervene and say “OK come on we’ve asked for quiet now, let’s quieten down”. I didn’t want to take over.* (Teacher)

Other teachers were very keen to intervene in sessions they believed were not going well. Facilitators often sensed a power struggle between themselves and the teacher, especially in the early stages. Some facilitators felt that they would have benefited from additional training in classroom management.

An issue that occurred repeatedly in the focus groups with young people related to their relationship with the facilitators. Some pupils were reticent about sharing their thoughts with facilitators they did not know well. A teacher also commented that it was difficult for a connection between facilitators and pupils to be formed over a relatively short period. Whilst every effort was made to ensure that the same pair of facilitators delivered the programme for the nine sessions in each class, due to practical constraints, this was not always possible. When facilitator pairings changed, it was harder for rapport and trust to develop between them and the pupils:

*It made it a bit awkward because we got to know the people and all of a sudden they were swapped.* (Year 8 male)

Some young people commented that they liked the facilitators, found them to be friendly, and felt that it was easier to talk to them about personal issues than their teachers. Pupils may feel uncomfortable about talking to teachers about mental health because they see them in a very different role. Developing a rapport between facilitators and young people was clearly important, even if the extent to which this was achieved was variable.

### 3.3. Flexibility and Differentiation

Suitability of the programme for different students was questioned, particularly with regard to age. Most teachers and facilitators felt that the classroom-based CBT was well pitched for the Year 8 pupils whom they considered to have grasped the concepts and applied them effectively:

*…the Lower School I think really enjoyed it.* (Teacher)

However, the programme was generally not considered to be age appropriate for older pupils, some of whom found it patronising or felt they had already learnt some of the skills. Most teachers who were involved in classroom-based CBT with older year groups felt that it was best suited to a younger age:

*I think it would have been much more successful if it had, for instance, been done with the Year 8 group, which is where I would have situated those particular materials.* (Teacher)

*I think with Year 7 this (classroom-based CBT) would have been fantastic and it’s very similar to the Year 7 scheme of work we already use.* (Teacher)

Likewise, the feedback from older pupils was not as positive. A Year 9 pupil reported that the hypothetical scenarios in the classroom-based CBT tackled subjects that were minor everyday issues for them and some teachers agreed that the role plays were not always targeted at topics that were relevant to the older pupils. There was a general feeling from facilitators and teachers that the older groups considered that they already knew the principles and content. One teacher believed that the classroom-based CBT was teaching skills that Year 11 pupils were already likely to have acquired.

Most facilitators and teachers felt that different versions of classroom-based CBT should have been available to better accommodate the specific needs of the different age groups involved in the study:

*All the sessions were the same... for Year 9 and 10 they were identical. It wasn’t age appropriate, I don’t think*. (Teacher)

*I think the idea of Key Stages is really important… we’re going in and aiming it at Year 8 who are 13 to Year 11 who are 16*. (Facilitator)

To maximise treatment fidelity, it was essential that facilitators delivered the programme in line with previous studies where RAP had been shown to be efficacious. Although there could be some flexibility in how materials were delivered, there was a need for consistency in the content. Some teachers were frustrated by what they perceived to be a lack of scope to innovate and not having been involved in developing the programme:

*There must be some flexibility built into it... your own guys were strangled by some of the rules that were so rigid.* (Teacher)

*If we’d have been consulted, you could have actually done loads with it*. (Teacher)

Advice about the materials had been sought from teachers in the pilot school and revisions made accordingly. Furthermore, the research team attempted to address teacher requests for a more interactive approach to sessions in the main study by adapting delivery but retaining the content and key messages. Some facilitators felt that involving individual teachers in each of the participating schools at the planning stage of the programme might have increased their willingness to contribute positively. However, on a practical level, involving individual teachers in development would be very resource intensive and the need for consistency and treatment fidelity when rolling out such programmes means that this issue would require careful consideration. 

### 3.4. Sustainability of Programme

For the programme to be sustainable, it would need to be adopted by schools. However, all of the teachers interviewed stated that it would not be possible to run the classroom-based CBT in its current format because of timetabling restrictions and budgetary constraints if the schools had to fund it:

*We don’t, unfortunately, think we can give it nine sessions, just because of the constraints of what else we have to put into the curriculum, sadly... but we’ve put five in I think.* (Teacher)

*I think the programme’s excellent, but there just isn’t that kind of money in a budget really to cover things like that.* (Teacher)

Some of the teachers could see value in selecting aspects of the programme and condensing it, although the evidence indicates that a programme shorter than 8 sessions may be less effective [5]:

*I think it’s the sort of programme that teachers would take and adapt.* (Teacher)

Certain teachers felt that classroom-based CBT could be successfully delivered by school staff if enough guidance and training was provided. However, it was said that the quality would depend on the type of teacher in the role and the degree of ownership that they had over the lesson plans:

*I don’t think there’s anything about the way it’s been structured or put together, the subject matter, that makes it difficult for a teacher to pick up and go with. But again, I think it’s down to that person*. (Teacher)

Evidence to date suggests that teacher-led programmes tend to be less effective than those led by people external to the school [5], and the issues highlighted in this analysis in relation to variation in skills, enthusiasm and treatment fidelity may well contribute to this. 

### 3.5. School Context

Two main themes emerged in relation to the school context; the value placed on PSHE as a subject and implementation of classroom-based CBT within a busy school environment.

#### 3.5.1. Value of PSHE

There was a feeling amongst some teachers that a negative ethos surrounds PSHE and that it is under-valued as a subject. They stated that examined subjects tend to take precedence within the timetable. PSHE is commonly taught by people who are not specialists in the field, and teachers show variable levels of commitment and different degrees of comfort in addressing relevant topics:

*That’s the sad thing about our PSHE, unfortunately we don’t have a designated team... it is squeezed on the timetable, it’s not given the proper priority it should be.* (Teacher)

*It’s always one of the least funded departments, PSHE.* (Teacher)

The standard PSHE curriculum is often compromised by the inclusion of other activities. For example, a teacher stated that it is difficult to keep older pupils engaged in PSHE when they are approaching examinations and wish to use the time for revision. A facilitator suggested that pupils might have been in a better mind-set to engage with classroom-based CBT if it had been run outside of PSHE, e.g., during tutor time:

*The fact that the RAP sessions were in PSHE, a lesson that isn’t taken particularly seriously, I think that really affected how people came into the class.* (Facilitator)

#### 3.5.2. School Environment

Schools are complex and busy organisations with many competing demands on time and resources. Information about the PROMISE Project was usually provided to the senior management teams and PSHE co-ordinators at schools by the research team so that this could be cascaded to individual teachers delivering PSHE. Presentations were also provided at assemblies and/or at the beginning of the assessment sessions as appropriate. Teachers were also given information sheets during assessment sessions which contained general background information about the project. Nonetheless, it was the general view of facilitators that teachers did not have adequate information about the project before it commenced. They questioned whether greater publicity about the project within each school might have resulted in better “buy-in”. Efforts had been made by facilitators to meet with teachers in each school to discuss the classroom-based CBT programme, but this was not always possible due to the competing demands on their time. A teacher agreed that the lack of time to set up a briefing meeting for staff to meet the facilitators affected the team building between the two parties:

*Because it’s that initial bit... we didn’t get the bonding side of it, we didn’t get the team building side of it... we did not have that luxury.* (Teacher)

Some facilitators felt that delivering classroom-based CBT sessions in schools following brief initial training was challenging and they would have benefited from a period of acclimatisation in schools prior to the start of their role. One of the facilitators commented that having some time beforehand to establish an effective working relationship with teachers might have helped to clarify roles and relieve some of the tensions that were experienced. Some facilitators also suggested that having additional time with teachers to plan sessions would have been helpful. Many teachers acknowledged the difficulties associated with achieving this, commenting that competing activities made it impossible to free up sufficient time to meet before or after lessons to discuss and review sessions.

Certain facilitators felt that communications within the schools needed to be improved. The research team did endeavour to communicate information to the schools via a key link person but messages were not always passed on to class teachers due to the complex nature of the school environment. For example, sometimes teachers did not know that the facilitators were coming and why, and essential equipment was not always set up, despite repeated requests:

*We needed the equipment there and it was really frustrating to turn up and the school go, “Oh, I didn’t know you needed a laptop”.* (Facilitator)

PSHE co-ordinators recognised that a project on the scale of PROMISE required an intense amount of commitment, which was difficult to sustain over a long period of time.

*I think the length of time that you’re here is quite challenging for us… it’s just trying to fit that in with our curriculum, it’s really hard.* (Teacher)

One facilitator recognised that implementing the programme in schools had proved to be immensely challenging:

*I think it’s difficult bringing mental health programmes into a school environment and it’s a massive job to integrate that kind of clinical into everyday school.* (Facilitator)

## 4. General Discussion

This qualitative evaluation provides some potential explanations why RAP, which has been efficacious in small scale studies, was not when rolled out under more diverse everyday conditions across several schools. Whilst some of the factors might be specific to the programme itself, others are more general and may explain why other classroom-based CBT depression prevention programmes have also been ineffective when implemented on a larger scale. As such, the findings highlight potential barriers to and opportunities for implementing and sustaining a mental health-related programme in schools. Key themes which emerged from the data were related to structure, content, delivery, flexibility, differentiation and sustainability of the programme, and the school context. 

As previously stated, engagement with and sustainability of school-based programmes is likely to be strongly influenced by teacher and pupil satisfaction [33] and a number of previous studies have reported that classroom-based CBT was positively received and perceived to be useful by teachers and young people [10,18,41]. The acceptability of classroom-based CBT in the PROMISE Project appeared to be reasonably good for Year 8 students, and the hands-on activities were particularly well received. However, acceptability for the older year groups was relatively poor, possibly because they had learnt some of the concepts before or that the materials were pitched at too young a level for them. Ensuring that such a programme is age appropriate is likely to become more difficult when attempting to roll it out across a large number of pupils with a wide age range.

An associated theme which emerged in this qualitative evaluation was the optimal age for the delivery of classroom-based CBT. There was no evidence in the quantitative analysis from the PROMISE Project to indicate that the effects of classroom-based CBT were associated with year group [23]. However, symptoms of depression were already common in the Year 8 students, indicating that it may be necessary to start prevention programmes at a younger age. It has been suggested that preventive interventions should occur before the general increase in depressive symptoms (around age 13–14 years) and continue during the time when rates and symptoms tend to increase (around ages 15–18 years) [42]. Others have suggested the optimal time for such a programme may actually be in late childhood at around age 9–10 years [3,43,44].

An alternative to programmes focusing on a specific age group are developmentally tailored programmes running across multiple school years [45]. Continuous and extended programmes have been recommended [46] and such approaches could help to cater appropriately for different age groups, as advocated by teachers and facilitators in this study. That said, the Beyondblue trial [19,20] was delivered over a three year period and was unsuccessful in preventing depression. Furthermore, it is important to bear in mind that such programmes are costly and require continued commitment from multiple agencies [18].

While some teachers were engaged and enthusiastic, others were negative about the programme, indicating that acceptability for teachers was variable. This negativity might have reflected a general scepticism about mental health-related programmes being delivered in classes. This could have been due to the research team not communicating effectively enough the nature of childhood depression and the rationale for using CBT in this context. The number of different teachers and classes involved in a large scale study inevitably makes effective communication more difficult to achieve. As previously stated, teachers in a study of RAP in New Zealand felt that they could deliver the programme more effectively if they were able to deviate from the manual and adapt the way some of the concepts were taught [9]. Although this may improve teacher engagement, this would likely compromise treatment fidelity as active components may be lost. 

The quality of delivery by facilitators was reported by teachers to be variable, and facilitators acknowledged that they found delivery challenging, particularly when new to delivering the intervention. Facilitators’ knowledge and confidence have been identified as important factors in relation to the effectiveness of prevention programmes [47]. Large scale studies utilising external support require many facilitators, and even if selected on similar criteria and identically trained to deliver the intervention, individual differences will contribute to how effectively this is undertaken.

Our findings indicated that the implementation of classroom-based CBT could have been facilitated by more initial communication and planning, which is a common issue for school based programmes [30]. In theory, addressing this should be relatively straightforward. Facilitators’ suggestions for tackling some of these issues included having a period of acclimatisation in schools and more training in classroom management, and finding more time for teachers and facilitators to work together and develop good relationships, plan and resolve issues. However, in practice, achieving this is extremely difficult as teachers have many demands on their time and often have to deal with urgent issues at school. To allow teachers to free up enough time to work with facilitators would have cost implications (e.g., providing backfill funding for a substitute teacher or paying for overtime) and may involve meeting away from the school site to ensure this time is protected. Whether this would be feasible and sustainable in reality is an issue that would need to be considered. 

It was strongly felt by teachers that the classroom-based CBT was too lengthy, resource-intensive, and costly in its current format to be routinely integrated into the PSHE curriculum. It has been argued that to enhance maintenance, shorter programmes should be considered [18]. However, between 8 and 12 sessions are considered optimal for depression prevention intervention effectiveness in schools [5], so dosage may be insufficient if programme length is reduced. Furthermore, the evidence to date suggests that teacher-led programmes are less effective than those led by other programme leaders [5]. Therefore, while shorter, more flexible, teacher-led programmes may be more sustainable and be easier to fit in to the school context, they may not be effective. School culture and context can also serve to inhibit or facilitate the implementation of classroom-based CBT in terms of head teacher support, teacher dynamics, and resource issues [17]. In the Beyondblue trial, the authors noted that embedding the programme within crowded school agendas and engaging young adolescents was problematic [19,20]. The qualitative findings of the current study highlight similar challenges. 

Teachers commented that PSHE was under-valued and under-resourced in UK schools and was typically not assigned the same level of importance as examined subjects. This is understandable given that UK government targets and school performance tables focus on academic attainment and managerial factors (e.g., workforce, finances) [48]. The lack of value placed on PSHE in general may have contributed to the lack of engagement with the classroom-based CBT. Given the potential for reaching a large proportion of the adolescent population in secondary schools and the importance of the topics covered in PSHE, the lack of value placed on this area of the curriculum seems to represent a wasted opportunity.

The findings suggest that, for depression prevention programmes to be successfully implemented in schools, a major cultural and policy shift would be required within the education system to ensure such programmes were valued, prioritised, and allocated sufficient time in the curriculum. This would place a greater emphasis on improving well-being and life skills, rather than focusing to such a great extent on academic achievement. However, a long period of time is required to implement policy and practice changes at whole-school levels [20]. So, although secondary schools provide a convenient location, they might not currently be a suitable context for delivering mental health prevention programmes. Helping children to develop mental health resilience skills within primary schools may be an option worth exploring as these environments are typically smaller, less complex, have more timetabling flexibility, and have a more nurturing and supportive focus. However, the issues with transporting efficacious prevention programmes to heterogeneous school settings would still need to be addressed.

### Study Strengths and Limitations

Due to the competing demands on the time of pupils and staff, it was not possible to randomly select pupils for the focus groups. The participants in this study were largely self-selecting (or teacher selected in the case of young people), which may have introduced response bias. Furthermore, it was not possible to elicit feedback from young people and teachers at every school. The fact that all interviews and focus groups were conducted by members of the PROMISE Project team could have resulted in participants feeling obliged to respond in a socially desirable way, although the interviews were carried out by researchers or the Trial Manager, rather than by facilitators who delivered the interventions. Nonetheless, views were gathered from a relatively large sample for a qualitative study (n = 70), with a cross-section of service providers and recipients across schools and year groups being represented, and a diverse range of views obtained. Thematic analysis enabled in depth interpretation of the transcripts and a rigorous verification strategy demonstrated the consistency of this interpretation across the three researchers. Furthermore, the fact that the process evaluation was conducted before the trial outcomes were known minimises associated biases.

## 5. Conclusions

This process evaluation has helped us to understand the possible reasons why this intervention, amongst others, failed to find an effect at the pragmatic large-scale stage of implementation.

Whilst schools provide a convenient setting for classroom-CBT programmes to prevent depression and allow good reach, the current study highlighted how challenging it can be in reality to deliver such programmes across several schools under everyday conditions. Although of primary concern to the implementation of the RAP programme in the school context in the UK, the findings have wider relevance to other similar trials. The difficulties in overcoming these challenges should not be underestimated as there is considerable pressure on resources in schools and it takes time to elicit change in school policy and culture. Based on the experiences of this study, it is considered that a number of issues need to be resolved before being able to confidently endorse the routine implementation of universal depression prevention programmes in the secondary school environment.
